# Emphysematous pyelonephritis with calculus: Management strategies

**DOI:** 10.4103/0970-1591.33718

**Published:** 2007

**Authors:** Tanmaya Goel, Sreedhar Reddy, Joseph Thomas

**Affiliations:** Department of Urology, Kasturba Medical College, Manipal, India

**Keywords:** Calculus, emphysematous pyelonephritis, nephrectomy, nephron salvage, percutaneous nephrolithotomy, percutaneous drainage, stenting

## Abstract

**Objective:**

Emphysematous pyelonephritis (EPN) with calculus is well recognized but with very few reports on its treatment. Our aim is to elucidate our experience in its successful management.

**Materials and Methods:**

Over four years, we diagnosed seven cases (eight renal units) of EPN, out of which two patients (three renal units) had EPN with urinary calculi. After the initial conservative management of EPN, the stones were tackled appropriately.

**Results:**

EPN was initially managed effectively with antibiotics and supportive care. Once the patient was stable, the stones were cleared in a step-wise fashion. The associated postoperative complications were also tackled efficiently with preservation of renal function.

**Conclusion:**

In EPN with stones, nephrectomy is not the sole option available and they can be effectively managed with open / endoscopic measures.

Emphysematous pyelonephritis (EPN) is a rare acute necrotizing infection of the kidneys[1] with presence of gas in the collecting system, renal parenchyma or the perirenal / pararenal spaces.[2] Though it occurs commonly in diabetics[3] or immunocompromized patients,[4] its association with calculus disease is well documented.[5–7] Emphysematous pyelonephritis in general has a fulminant course, as most cases are recognized late.[8] Prompt recognition and management is the key to survival. Appropriate staging of the disease[2] and identification of the adverse prognostic factors like elevated serum creatinine, shock, thrombocytopenia or altered sensorium are imperative for its successful management. With advances in imaging modalities and antibiotics, diagnosis can be made at an earlier stage and managed aggressively. Adequate control of sugars with resuscitative and supportive care is responsible for the overall decreased mortality rates.

The surgical management was limited to nephrectomy earlier.[9,10] This had its own pitfalls—of performing surgery in an unfit / sick patient. There is even a possibility of making the patient anephric with dependence on dialysis. The advancement in endourology has brought forth an attractive and effective option of percutaneous drainage of the abscess[11] and /or stent drainage.[12] These along with good antibiotic coverage, has resulted in increased renal salvage even in patients with bilateral EPN[13,14] or solitary kidneys;[15] nephrectomy being indicated only for poor responders after adequate attempts at stabilization.[16,17] The management of the offending calculus needs to be tailored according to the health status of the patient once the acute crisis is tided over.

## MATERIALS AND METHODS

We present our experience in the management of stone disease in cases of EPN which were undertaken over a period of four years. We had seven cases (eight renal units) of EPN diagnosed at our center, of which four were females. Diabetes was the predisposing feature in 50% of the patients with one detected at the time of presentation with EPN. Two patients presented with acute renal failure and two with hypotension, tachycardia and altered sensorium. One patient had bilateral EPN. Out of the seven patients, two patients (three renal units) had features of EPN with renal / ureteric calculi.

## RESULTS

The initial line of management included prompt diagnostic imaging with computerized tomography (CT) scan, institution of parenteral antibiotics and supportive care. Primary percutaneous drainage or double ‘J’ (DJ) stenting was done to stabilize the patient.

The five patients of EPN without calculi were managed conservatively with oxygen inhalation, intravenous fluids, antibiotics, sugar control, electrolyte and acidosis correction. Drainage was instituted in the form of either DJ stenting (one patient) or PCN (one patient). Only one patient who had presented with acute renal failure had to be initiated on hemodialysis. All the patients improved symptomatically and radiologically. Nephrectomy could be avoided in all these patients.

**Figure 1:**
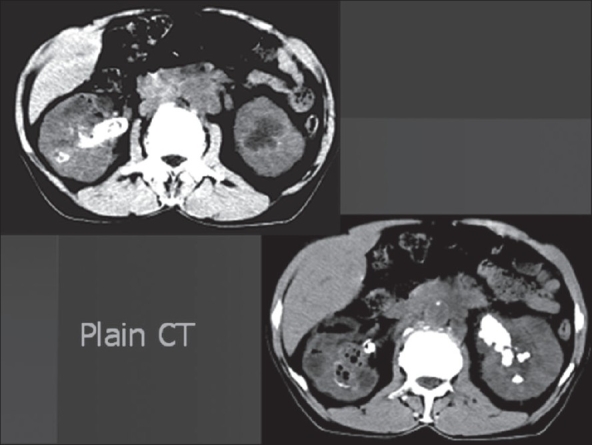
Computerized tomogram exhibiting bilateral EPN with renal calculi

**Figure 2:**
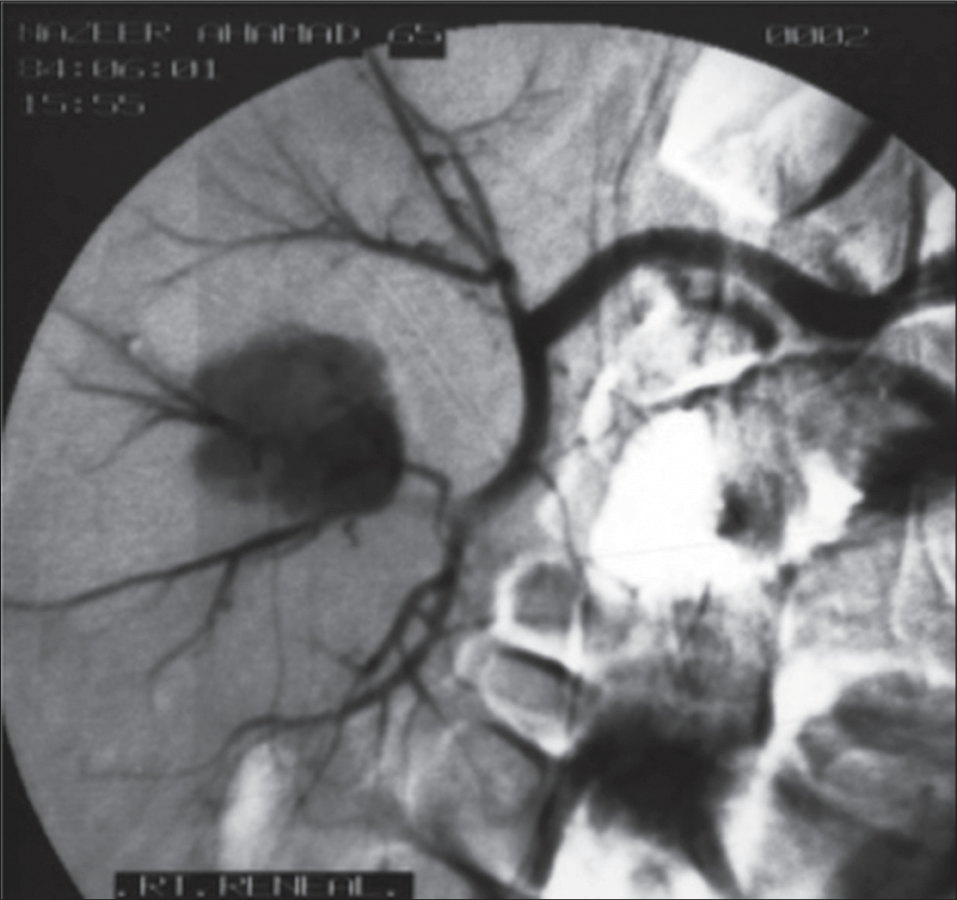
Selective right renal angiogram depicting the bleeding vessel

Among the two patients of EPN with stone, the first patient, a 64-year-old male, had bilateral renal stones with bilateral EPN (Class 4) [Figure 1] and renal failure with a serum creatinine of 4.5mg/dl. Supportive care and intravenous antibiotics (third generation cephalosporins) were initiated. The patient underwent plain CT scan and was subjected to bilateral double ‘J’ stenting. Urine culture grew *Klebsiella* species (10^5^ cfu/ ml). The renal failure improved and serum creatinine values stabilized at 2.2 mg/dl. After six weeks, he underwent right percutaneous nephrolithotomy (PCNL) through an inferior calyceal puncture. A nephrostomy tube and DJ stent were kept. On the fifth postoperative day, he had hematuria with spiking pyrexia. In spite of higher antibiotics and eight units of blood transfusion, the patient continued to have bleeding. Selective right renal angiography [Figure 2] was done, bleeding site identified and embolized. After a month, he underwent left extended pyelolithotomy. Bilateral stents were removed after another six weeks. The patient has been on regular follow-up for the last three years with repeated biochemistry, ultrasonography (USG) and DTPA renograms. He has a serum creatinine which leveled at 1.8-2.0 mg/ dl.

**Figure 3:**
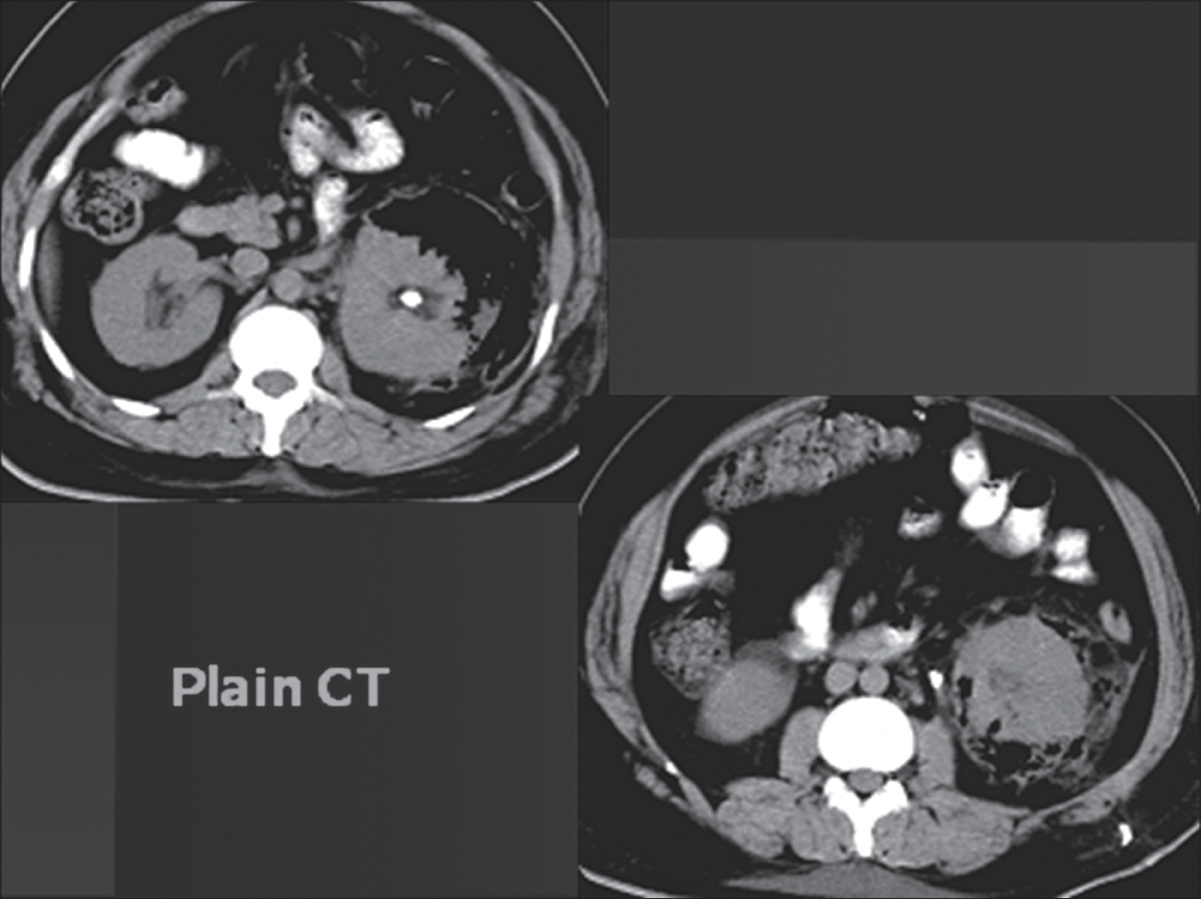
Computerized tomogram showing left EPN with renal and upper ureteric calculi

The second patient, a 42-year-old male, had EPN with a left renal and an upper ureteric calculus. He was in ketoacidosis with high blood sugars, detected for the first time. He was hydrated, started on oxygen inhalation, insulin infusion along with third generation cephalosporins and aminoglycosides. He underwent plain and contrast enhanced CT abdomen (after confirming normal serum creatinine levels) which showed Class 3 EPN [Figure 3]. Emergency percutaneous drainage of the abscess and double ‘J’ stenting were done. It drained thick, foul-smelling pus. Though the urine grew *E. coli* (10^5^ cfu/ml), the pus culture was sterile. The drainage tube was removed after two weeks. The patient underwent left PCNL four weeks later. Retrograde pyelography at that time showed a serpiginous tract from the upper calyx to the earlier drainage site. The upper ureteric calculus was pushed back into the kidney and both the stones were removed via a middle calyceal puncture. The double ‘J’ stent was retained. The granulation tissue at the previous drain site was excised along with the tract. Postoperative period was uneventful and the patient recovered without any complication. At one-year follow-up with USG and CT scan, the kidney is scarred; but with a good functional status and no recurrence of stone. The patient profile, treatment procedures, complications encountered and their management and follow-up undertaken are summarized in Table 1.

**Table 1:** Patient profile, presentation, management, complications and follow-up

No.	Patient	Site of calculus	Management	Complications	Follow-up at three month
1.	64-year-old male, non- diabetic, elevated creatinine	Bilateral renal	Rt PCNL Lt Pyelolithotomy	Hemorrhage - Angioembolization	*Creatinine levelled at 1.8 mg/dl
2.	42-year-old male, diabetic, normal creatinine	Lt renal and upper ureteric	Lt PCNL	Nephrocutaneous fistula - excision	*Diabetes controlled*Normal renal function*Scarred left kidney

PCNL - Percutaneous nephrolithotomy

Thus, in all our cases of EPN with calculi, after the initial conservative management with antibiotics and drainage, the stones were effectively managed with open or endoscopic measures with nephron salvage and a good functional outcome.

## DISCUSSION

The role of conservative medical management of EPN with antibiotics and supportive care along with percutaneous drainage/ stenting as a treatment option is not disputed today.[18,19] But there is not much literary evidence with regard to stone management in EPN kidneys. There are case reports mentioning the possibility of a retrograde procedure or PCNL through the access which was established initially for drainage.[20] However, these reports are not forthcoming about the timing, problems encountered, complications and final outcome of the interventions undertaken for stone clearance.

Our experience elucidates the importance of initial conservative management of EPN along with drainage, followed by management of calculus in a graded fashion. Such a staged approach is the cornerstone of treatment. Complication rates were similar to those encountered with regular PCNL. The bleeding encountered in one of the cases was tackled effectively with angioembolization. The nephrocutaneous fistula which developed in the second case due to the parenchymal necrosis was also managed efficaciously.

## CONCLUSIONS

From our limited experience, we can safely deduce that the standard practice of nephrectomy as the sole option available for EPN in a renal unit with calculus should be reviewed. The calculi can be effectively managed with open or endoscopic measures with renal preservation and a good functional outcome.
